# Factors associated with unsuccessful tuberculosis treatment in Manaus, Amazonas, from 2011 to 2021

**DOI:** 10.1590/1980-220X-REEUSP-2023-0431en

**Published:** 2024-10-07

**Authors:** Felipe Alves de Almeida, Maria Jacirema Ferreira Gonçalves

**Affiliations:** 1 Universidade Federal do Amazonas, Escola de Enfermagem de Manaus, Programa de Pós-Graduação em Enfermagem, Manaus, AM, Brazil.; 2 Universidade Federal do Amazonas, Escola de Enfermagem de Manaus, Departamento de Enfermagem, Manaus, AM, Brazil.

**Keywords:** Tuberculosis, Resultado del Tratamiento, Cumplimiento de la Medicación, Vulnerabilidad en Salud, Perdida de Seguimiento, Tuberculosis, Resultado del Tratamiento, Cumplimiento de la Medicación, Vulnerabilidad en Salud, Perdida de Seguimiento

## Abstract

**Objective:**

To identify vulnerability factors associated with unsuccessful tuberculosis treatment outcomes between 2011 and 2021 in Manaus, Amazonas.

**Method:**

Ecological study using tuberculosis case notification data from the Notifiable Diseases Information System, from 2011 to 2021, of residents in Manaus. The variables refer to treatment outcomes and patient vulnerability, according to the theoretical model: individual, programmatic and social. The analysis tested the association between vulnerability and tuberculosis treatment non-success, measured by the occurrence of death, loss to follow-up or treatment default. The Odds Ratio estimate with confidence interval was obtained by logistic regression, according to a hierarchical model.

**Results:**

The following factors were more likely to lead to unsuccessful tuberculosis treatment: individual vulnerability (age group 20 to 29 years and over 60 years, indigenous race, HIV+, drug use); programmatic vulnerability (not having an HIV test), social vulnerability (special population).

**Conclusion:**

Individual vulnerability was more strongly associated with non-success. Intervention is needed to explore the points of greatest individual vulnerability, enabling effective action to prevent unsuccessful tuberculosis treatment.

## RESUMEN

**Objetivo:**

Identificar los factores de vulnerabilidad asociados con el fracaso del tratamiento de la tuberculosis entre 2011 y 2021 en Manaos, Amazonas.

**Método:**

Estudio ecológico a partir de datos de notificación de casos de tuberculosis del Sistema de Información de Enfermedades de Declaración Obligatoria, de 2011 a 2021, de residentes en Manaos. Las variables se refieren a los resultados del tratamiento y la vulnerabilidad de los pacientes, de acuerdo con el modelo teórico: individual, programático y social. El análisis evaluó la asociación entre la vulnerabilidad y el fracaso del tratamiento de la tuberculosis, medido por la ocurrencia de fallecimiento, pérdida de seguimiento o fracaso del tratamiento. La estimación del Odds Ratio con intervalo de confianza se obtuvo por regresión logística, según un modelo jerárquico.

**Resultados:**

Los siguientes factores fueron asociados al fracaso del tratamiento de la tuberculosis: vulnerabilidad individual (grupo de edad de 20 a 29 años y mayores de 60 años, raza indígena, VIH+, consumo de drogas); vulnerabilidad del programa (no realizar la prueba del VIH), vulnerabilidad social (población especial).

**Conclusión:**

La vulnerabilidad individual se asoció más fuertemente con el fracaso. Es necesario intervenir para explorar los puntos de mayor vulnerabilidad individual, permitiendo una acción eficaz para prevenir el fracaso del tratamiento de la tuberculosis.

## INTRODUCTION

The World Health Organization (WHO) has acknowledged the significance of Tuberculosis (TB) in the world as a public health problem and highlights 30 countries that concentrate around 90% of the disease’s burden, including Brazil, that recorded 80,012 new cases of TB (37.3 cases per 100,000 inhabitants) and 5,845 deaths (2.8 deaths per 100,000 inhabitants) for the year 2022. In the same year, Amazonas reported 3,548 new cases (81.6 cases per 100,000 inhabitants) and 218 deaths (5.1 deaths per 100,000 inhabitants). Manaus recorded 2,512 new cases (113.2 cases per 100,000 inhabitants) and 134 deaths (5.9 deaths per 100,000 inhabitants). Amazonas had the second highest TB incidence and mortality rate in 2023; Manaus stands out as the capital with the highest incidence and the second highest mortality rate^(1)^.

Despite this critical condition, the disease has the potential to be cured and its treatment has been a strategic target for control. Patient follow-up is carried out at primary care level and has a prognosis for cure when carried out correctly^(2)^. However, it is a long treatment, which can cause adverse reactions and, in some cases, there may be difficulties in accessing the health service, as well as interfering with the patient’s social and individual issues, generating possibilities for unsuccessful treatment^(3)^.

Unsuccessful treatment increases the incidence of cases, raises the risk of drug resistance, increases the chances of morbidity and mortality and raises costs for the public sector^(3)^. In this study, unsuccessful treatment includes the following outcomes: death, treatment failure and treatment default. The latter term is still predominant in the literature and is recorded as such in the information system, but it is recommended to use “loss to follow-up” of TB treatment, since it includes the various elements involved in this outcome, and avoids blaming the patient for quitting treatment^(4)^.

Several factors are identified in the literature as being associated with TB treatment outcomes in isolation. The factors associated with TB cure are: being female, having more than 9 years of education, being white race, undergoing Directly Observed Treatment (DOT) and others^(5)^. As for the main unfavorable outcomes, loss to follow-up and death from TB, the associated factors are age older than or equal to 60 years, non-white race, HIV-positive result, pulmonary form, among others^(6,7)^. However, in general, these factors are observed in the literature in a Cartesian way, disregarding the vulnerability scenarios that they can generate, exerting an influence on each other, and together, on the outcome. In this paper, the term vulnerability is understood as the fragility of groups or individuals to have their needs met and this vulnerability is made up of the mutual influence of multiple factors^(8)^.

So, despite the consistent theoretical framework that has been built up over the years about TB, coping with the disease still remains a challenge in Manaus. It should be noted that Manaus has unique social and geographical conditions in comparison to other locations in the country. It is inferred that these conditions can interfere in the factors that influence the outcomes of TB treatment, generating scenarios of vulnerability specific to the locality that need to be studied. In addition, the studies produced are concerned with evaluating the outcomes in a partitioned manner, analyzing treatment default or cure individually, and ignore the other outcomes^(9)^.

Therefore, with the aim of understanding the outcomes of TB treatment in Manaus, in order to look at this scenario from a more holistic perspective and to provide support for health and nursing in Amazonas, this study aims to identify the vulnerability factors associated with unsuccessful TB treatment outcomes from 2011 to 2021 in the city of Manaus, Amazonas.

## METHOD

### Type of Study

Epidemiological, observational, ecological and analytical study, with retrospective collection of annual notifications of new TB cases from 2011 to 2021 in Manaus, Amazonas. The recommendations of the Strengthening the Reporting of Observational Studies in Epidemiology (STROBE) checklist were used, adapted to the type of study^(10)^.

### Study Site

Manaus, the capital of Amazonas, is 11,401,092km^2^ in size and represents 52.5% of the population of Amazonas, with an estimated 2,255,903 inhabitants in 2021. In 2022, it concentrated more than 70% of the state’s TB cases, making it the capital with the second highest TB incidence rate in Brazil^(11)^.

### Population and Selection Criteria

The reference population was considered to be all new cases of TB in Manaus residents notified to the Notifiable Diseases Information System (SINAN in the Portuguese acronym) between 2011 and 2021. The inclusion criteria were as follows: new, unknown and post-mortem cases, all clinical forms, with the condition that the closure records were complete in the database, updated until December 2022. Duplicate cases, changes of diagnosis and transfers were excluded from the analysis, due to the impossibility of correctly assessing the outcome of unsuccessful treatment.

### Sample Definition

We considered the entire universe of cases that met the above criteria, living in Manaus, notified between 2011 and 2021.

### Study Protocol

We used secondary data on TB notifications in Manaus from the municipal SINAN database, which was made available in an anonymized form by the Municipal Health Department and updated in May 2023. which made it possible to analyze more recent data.

To select the variables, we used the TB notification form and the concept of vulnerability^(8)^. This is expressed as the fragility of individuals or groups to have their needs met, looking for elements associated or associable with the process of becoming ill, not becoming ill and coping. Therefore, according to the concept, the health-disease process involves the interaction of a set of aspects, adopting the vulnerability dimension approach. The independent variables were then organized into blocks aligned with the dimensions of vulnerability:

– individual vulnerability (characteristics of the individual): sex; age group; race; alcohol, tobacco or other drugs; diabetes; mental illness; other conditions (any other condition reported or identified in the patient and recorded as other in SINAN, such as arthritis, arthrosis and systemic arterial hypertension); clinical form and bacilliferous pulmonary case;
– programmatic vulnerability (aspects of the TB control program): offer of HIV testing; directly observed treatment (DOT); offer of chest X-ray; diagnostic test (sputum smear microscopy or rapid molecular test);
– social vulnerability (social context of the individual): social vulnerability: special population (homeless population, population deprived of liberty, health professionals, immigrants, population living in institutions); education (in years of study, which in this case, the age group < 15 years was considered as ‘not applicable’, due to the impossibility of having completed studies); and, beneficiary of a government cash transfer program.

The dependent variable considered TB treatment outcomes: cure, loss of TB treatment follow-up, death from TB, death from other causes, other outcomes (treatment failure, drug-resistant TB (DR-TB) and change of regimen) and unsuccessful treatment (loss of TB treatment follow-up, death from TB, death from other causes and treatment failure). These outcomes were obtained from the “closure status” field of the notification data.

The variable “death from other causes” was included in the study because it was impossible to ascertain what the outcome would have been if the cases had not died, although they would have had the same chance of any of the outcomes studied. Another issue that cannot be controlled is whether TB was consciously excluded as the cause of death when filling out the death certificate, and since this is secondary data, there is no way of verifying it, given that TB death surveillance was implemented in Manaus in 2017^(12)^. In addition, the ‘other outcomes’ category was grouped together for two reasons: low frequency and because they are generally the result of previous treatment interruption. The unsuccessful treatment variable was grouped in the above way to contrast with cure, which would be the success of the treatment, and to broaden the binary understanding of cure and loss to follow-up, since in reality there are other outcomes that need to be analyzed.

### Data Analysis and Processing

All the data was compiled and analyzed using the statistical software R version 4.1.3 and the following packages were used in the program: magrittr, readlx, tidyverse, dplyr, tidyr, lubridate, epiDisplay, foreign, r companion, carData and gtsummary. In the descriptive analysis, absolute and percentage frequencies were presented for the categorical variables, exploring their relationship with unsuccessful TB treatment and with each outcome. This last aspect was analyzed using Pearson’s ­chi-square, considering statistical significance < 5% and a 95% confidence interval. In order to identify categories of association within each variable, the standardized residual of the chi-square was analyzed, and residuals >1.96 were considered significant at the 5% level. The magnitude of each significance was assessed using Cramer’s V Coefficient, which quantifies the intensity of the relationship between the observed outcome and the associated factor. This coefficient varies between 0 and 1, with values closer to 1 indicating greater intensity of the effect.

The bivariate logistic analysis tested the relationship between the independent variables and the non-success outcome. For multivariable logistic regression analysis, hierarchical modeling was performed^(13)^, according to the proximity of the vulnerability blocks to the outcome, considering the conceptual model proposed. The conceptual model of vulnerability is based on the social determinants of health, which determines the proximity of factors influencing the development of the health-disease process. Proximal factors are inherent to individuals; intermediate factors concern life and everyday life; and distal factors refer to the economic and governmental macro-structure. In this study, individual, programmatic and social factors were considered as proximal, intermediate and distal, respectively, in the hierarchical modeling^(8,13,14)^. The variables that obtained a P-value <0.20 in the bivariate analysis were grouped into the respective vulnerability block; they were then inserted into the hierarchical model considering the blocks according to their theoretical proximity to the outcome: individual vulnerability, programmatic vulnerability and social vulnerability. The result is expressed as an odds ratio (OR) with a 95% confidence interval. The quality of the hierarchical model was assessed using the Akaike information criterion (AIC) and the final model was selected using the variance reduction analysis with a P-value < 0.05.

### Ethical Aspects

The study was submitted to and approved by the Research Ethics Committee of the Federal University of Amazonas (CEP/UFAM) in 2023 under opinion 5,984,601, in accordance with Resolution No. 466/12 of the National Health Council (CNS), ensuring that data pertaining to the identity of the subjects will remain confidential. As this is a secondary data study, the free and informed consent form was waived.

## RESULTS

This study analyzed 25,754 TB cases notified in Manaus between 2011 and 2021. which met the established inclusion criteria. Table 1 shows the distribution of vulnerability aspects and their association with TB treatment outcomes. For the variables in the individual vulnerability block, the following significant associations for each outcome are highlighted:

**Table 1:** Distribution of new cases of all forms of tuberculosis, in vulnerability blocks, according to treatment outcomes – Manaus, AM, Brazil, 2011 to 2021.

Variables	Cure 18640 (72.4)		Loss to follow-up of TB treatment 3987 (15.5)		TB deaths 1415 (5.5)		Death by other causes 846 (3.3)		Other outcomes 866 (3.4)		V-Cramer test
	N	%	N	%	N	%	N	%	N	%	
Individual vulnerability	(SR)		(SR)		(SR)		(SR)		(SR)		P-value
*Sex*											
Male	10976	58.9	2834	71.1	563	66.5	972	68.7	496	57.3	V = 0.099
	(–14.01)		**(13.51)**		**(3.06)**		**(5.71)**		**(2.6)**		**<0.001**
Female	7664	41.1	1153	28.9	283	33.5	443	31.3	370	42.7	
	**(14.01)**		(–13.51)		(–3.06)		(–5.71)		(–2.6)		
*Age range (years)*											
<15	1039	5.6	154	3.9	28	3.3	41	2.9	15	1.7	V = 0.109
	**(7.37)**		(–3.47)		(–2.25)		(–3.67)		(–4.45)		**<0.001**
15–19	1596	8.5	391	9.8	25	2.9	22	1.6	47	5.4	
	**(4.59)**		**(4.35)**		(–5.56)		(–9.27)		(–2.91)		
20–29	4559	24.5	1349	33.8	102	12.1	258	18.2	206	23.8	
	(–4.07)		**(13.77)**		(–8.92)		(–6.16)		(–0.93)		
30–39	3647	19.6	931	23.3	104	12.3	337	23.8	191	22.1	
	(–4.3)		**(5.34)**		(–5.84)		**(3.45)**		(1.36)		
40–49	2926	15.7	571	14.3	118	13.9	250	17.7	129	14.9	
	(1.36)		(–2.25)		(–1.27)		**(2.31)**		(–0.51)		
50–59	2441	13.1	293	7.4	120	14.2	205	14.5	114	13.2	
	**(6.13)**		(–10.39)		(1.68)		**(2.55)**		(0.77)		
≥ 60	2432	13	298	7.5	349	41.3	302	21.3	164	18.9	
	(–5.41)		(–12.54)		**(23.6)**		**(8.51)**		**(4.49)**		
*Race*											
White	2256	12.1	325	8.1	74	8.8	141	10	82	9.5	V = 0.034
	**(7.65)**		(–6.59)		(–2.28)		(–1.49)		(–1.62)		**<0.001**
Black	588	3.1	160	4	22	2.6	47	3.3	24	2.8	
	(–1.62)		**(2.89)**		(–1.11)		(0.12)		(0.83)		
Yellow	106	0.6	24	0.6	1	0.1	8	0.6	1	0.1	
	(0.89)		(0.55)		(–1.71)		(0.11)		(–1.74)		
Brown	15068	80.8	3264	81.9	711	84	1175	83	731	84.4	
	(–3.37)		(0.92)		**(2.05)**		(1.68)		**(2.36)**		
Indigenous	128	0.7	43	1.1	10	1.2	15	1.1	9	1	
	(–3.2)		**(2.18)**		(1.28)		(1.15)		(0.82)		
Ignored	484	2.7	171	4.3	28	3.3	29	2	19	2.2	
	(–3.53)		**(5.8)**		(0.77)		(–1.92)		(–1.22)		
*HIV*											
positive	2176	11.7	632	15.8	78	9.2	808	57.1	180	20.8	V = 0.290
	(–24.46)		(1.55)		(–4.82)		**(45.53)**		**(4.81)**		**<0.001**
negative	16464	88.3	3355	84.2	768	90.8	607	42.9	686	79.2	
	**(24.26)**		(–1.55)		**(4.82)**		(–45.53)		(–4.81)		
*Use of alcohol, tobacco or other drugs*											
Yes	2553	13.7	1122	15.8	190	22.5	326	23	226	26.1	V = 0.152
	(–23.81)		**(20.03)**		**(4.16)**		**(6.04)**		**(7.1)**		**<0.001**
No	16087	86.3	2865	84.2	656	77.5	1089	77	640	73.9	
	**(23.81)**		(–20.03)		(–4.16)		(–6.04)		(–7.1)		
*Diabetes*											
Yes	1948	10.4	254	6.4	118	13.9	119	8.4	125	14.4	V = 0.062
	**(4.29)**		(–8.22)		**(3.94)**		(–2)		**(4.48)**		**<0.001**
No	16692	89.6	3733	93.6	728	86.1	1296	91.6	741	85.6	
	(–4.29)		**(8.22)**		(–3.94)		**(2)**		(–4.48)		
*Mental Illness*											
Yes	192	1	42	1	27	3.2	28	2		0.7	V = 0.04
	(–2.28)		(–0.59)		**(5.69)**		**(3.03)**		(–1.27)		**<0.001**
No	18448	99	3945	99	819	96.8	1387	98	860	99.3	
	**(2.82)**		(0.59)		(–5.69)		(–3.03)		(1.27)		
*Other diseases*											
Yes	1152	6.2	226	5.7	150	17.7	171	12.1	136	15.7	V = 0.113
	(–9.54)		(–3.89)		**(12.19)**		**(7.46)**		**(9.98)**		**<0.001**
No	17488	93.8	3761	94.3	696	82.3	1244	87.9	730	84.3	
	**(9.54)**		**(3.89)**		(–12.19)		(–7.46)		(–9.98)		
*Clinical presentation*											
Pulmonary	15369	82.4	3415	85.7	647	76.5	918	64.9	672	77.6	V = 0.093
	**(5.56)**		**(7.15)**		(–3.93)		(–16.73)		(–3.11)		**<0.001**
Extrapulmonary	2606	14	408	10.2	149	17.6	321	22.7	132	15.2	
	(–0.45)		(–7.53)		**(3.04)**		**(9.63)**		(1.04)		
Mixed (Pulmonary + Extrapulmonary)	665	3.6	164	4.1	50	5.9	176	12.4	62	7.2	
	(–9.81)		(–0.75)		**(2.28)**		**(15.39)**		**(4.15)**		
*Bacilliferous*											
Yes	11470	61.5	2797	70.2	469	55.4	490	34.6	618	71.4	V = 0.153
	(0.07)		**(12.19)**		(–3.7)		(–21.39)		**(6.06)**		**<0.001**
No	7170	38.5	1190	29.8	377	44.6	925	65.4	248	28.6	
	(–0.07)		(–12.19)		**(3.7)**		**(21.39)**		(–6.06)		
**Programmatic vulnerability**											
*HIV test performed*											
Yes	14110	75.7	2667	66.9	438	51.8	1100	77.7	654	75.5	V = 0.117
	**(12.05)**		(–10.54)		(–14.69)		**(3.59)**		(1.27)		**<0.001**
No	4530	24.3	1320	33.1	408	48.2	315	22.3	212	24.5	
	(–12.05)		**(10.54)**		**(14.69)**		(–3.59)		(–1.27)		
*Directly Observed Treatment (DOT)*											
Yes	2417	13	438	11	165	19.5	154	10.9	34	3.9	V = 0.065
	**(4.02)**		(–3.06)		**(6.31)**		(–1.84)		(–7.73)		**<0.001**
No	16223	87	3549	89	681	80.5	1261	89.1	832	96.1	
	(–4.02)		**(3.06)**		(–6.31)		(1.84)		**(7.73)**		
*X-ray*											
Yes	14475	77.7	2853	71.6	721	85.2	1267	89.5	674	77.8	V = 0.094
	(0.23)		(–9.99)		**(5.4)**		**(11.07)**		(0.15)		**<0.001**
No	4165	22.3	1134	28.4	125	14.8	148	10.5	192	22.2	
	(–0.23)		**(9.99)**		(–5.4)		(–11.07)		(–0.15)		
*Diagnostic examination (bacilloscopy or molecular test)*											
Yes	16256	87.2	3544	88.9	699	82.6	1048	74.1	784	90.5	V = 0.096
	**(3.84)**		**(4.41)**		(–3.56)		(–14.41)		**(3.37)**		**<0.001**
No	2384	12.8	443	11.1	147	17.4	367	26.9	81	9.5	
	(–3.84)		(–4.41)		**(3.56)**		**(14.41)**		(–3.37)		
**Social vulnerability**											
*Special population*											
Yes	1173	6.3	425	10.7	79	9.3	113	8	62	7.2	V = 0.063
	(–9.03)		**(9.22)**		**(2.46)**		(1.19)		(–0.04)		**<0.001**
No	17467	93.7	3562	89.3	767	90.7	1302	92	802	92.8	
	**(9.03)**		(–9.22)		(–2.46)		(–1.19)		(0.04)		
*Education (years or schooling)*											
Illiterate	320	1.7	75	1.9	64	7.6	50	3.5	29	3.3	V = 0.078
	(–6.76)		(–1)		**(11.32)**		**(3.91)**		**(2.64)**		**<0.001**
1–4 years	2663	14.3	583	14.6	162	19.1	220	15.6	133	15.4	
	(–2.33)		(0.04)		**(3.81)**		(1.03)		(0.64)		
5–9 years	3577	19.2	961	24.1	130	15.4	276	19.5	173	20	
	(–4.42)		**(7.29)**		(–3.34)		(–0.35)		(0.08)		
More than 10 years	7680	41.2	1208	30.3	188	22.2	501	35.4	393	45.4	
	**(13.28)**		(–11.86)		(–10.01)		(–2.63)		**(4.1)**		
Ignored	3359	18	1004	25.2	274	32.4	327	23.1	123	14.2	
	(–11.3)		**(9.37)**		**(9.39)**		**(3.26)**		(–4.17)		
Not applicable	1041	5.6	156	3.9	28	3.3	41	2.9	15	1.7	
	**(7.3)**		(–3.35)		(–2.26)		(–3.7)		(–4.46)		
*Beneficiary of government cash transfer program*											
Yes	889	4.8	205	5.1	66	7.8	81	5.7	43	5	V = 0.026
	(–2.58)		(0.49)		**(3.83)**		(1.31)		(–0.03)		**0.0013**
No	1775	95.2	3782	94.9	780	92.2	1334	94.3	823	95	
	**(2.58)**		(–0.49)		(–3.83)		(–1.31)		(0.03)		

Source: Notifiable Diseases Information System (SINAN), Manaus Municipal Health Department. Data obtained on 09/04/2023.

Notes: SR = Standardized residual – The percentage adds up to 100% in the column and chi–squared residuals larger than 1.96 are in bold, corresponding to the significance level for excess occurrences.

(1) cure: female sex, age group under 20 years and 50 to 59 years, white race, HIV negative, not using alcohol, tobacco or other drugs, diabetes, absence of mental illness or other conditions and, pulmonary form;
(2) loss to follow-up of TB treatment: male sex, 15 to 39 age group, black, indigenous or unknown race, use of alcohol, tobacco or other drugs, absence of diabetes or other conditions, pulmonary form and bacilliferous;
(3) death from TB: male sex, aged 60 or over, brown race, HIV-negative, use of alcohol, tobacco or other drugs, pulmonary, extrapulmonary or non-bacilliferous clinical form, and presence of diabetes, mental illness and other conditions;
(4) death from other causes: age group 30 and over, HIV positive, use of alcohol, tobacco or other drugs, mental illness and other conditions, extrapulmonary and mixed clinical forms, and non-bacilliferous cases; and
(5) other outcomes: age group greater than or equal to 60 years, brown race, HIV positive; use of alcohol, tobacco or other drugs, diabetes and other conditions, mixed clinical form and bacilliferous cases.

In the programmatic vulnerability block, the following significant associations were observed:

(1) cure: performing an HIV test, performing DOT and performing a diagnostic test;
(2) loss of TB treatment follow-up: no HIV test, no DOT, no X-ray and no diagnostic test;
(3) death from TB: no HIV test, no DOT, no X-ray and no diagnostic examination;
(4) death from other causes: HIV test, X-ray and no diagnostic examination; and
(5) other outcomes: no DOT and no diagnostic test.

As for the social vulnerability block, the significant associations for each outcome were:

(1) cure: not being a special population, education level greater than or equal to 10 years or not applicable, and not being a beneficiary of the government cash transfer program;
(2) loss to follow-up of TB treatment: being a special population, having between 5 and 9 years of education and no education;
(3) death from TB: special population, illiterate, less than 4 years of education and unknown education, and beneficiary of the government cash transfer program;
(4) death from other causes: illiterate and unknown education; and
(5) other outcomes: illiterate education and greater than or equal to 10 years of education.

The strongest effects are in the variables: age group, people living with HIV, use of alcohol, tobacco or other drugs, other health problems, bacilliferous cases and HIV testing (Cramer’s V test) (Table 1).

Other conditions represent any other condition reported or identified in the patient and recorded as other in SINAN, such as arthritis, arthrosis and systemic arterial hypertension. Special population includes homeless population, population deprived of liberty, health professionals, immigrants, population living in institutions.

Table 2 shows the results of the bivariate and multivariable logistic regression analysis according to the hierarchical model. Considering the limitations, due to the possibility of interaction and the problems with filling in the data, the education variable was not included in the multivariable model.

**Table 2:** Simple and multivariable logistic regression models for factors associated with unsuccessful tuberculosis treatment, according to hierarchical blocks of individual, programmatic and social vulnerability – Manaus, AM, Brazil, 2011 to 2021.

Variables	Crude analysis			Adjusted analysis					
Individual vulnerability				Model 1*		Model 2*		Model 3*	
	%	OR*	95%CI*	OR*	95%CI*	OR*	95%CI*	OR*	95%CI*
*Sex*									
Female	38.4	1		1		1		1	
Male	61.6	1.62	1.52–1.72	1.36	1.28–1.45	1.37	1.28–1.46	1.37	1.28–1.46
*Age range (years)*									
<15	5.1	1		1		1		1	
15**–**19	8.2	1.28	1.07–1.53	1.19	0.99–1.43	1.76	1.46–2.13	1.81	1.50–2.20
20**–**29	25.2	1.75	1.50–2.04	1.33	1.14–1.57	2.03	1.72–2.40	2.04	1.72–2.41
30**–**39	20.2	1.76	1.50–2.06	1.22	1.03–1.44	1.82	1.53–2.16	1.84	1.55–2.19
40**–**49	15.5	1.5	1.27–1.76	1.10	0.93–1.30	1.60	1.35–1.92	1.63	1.37–1.95
50**–**59	12.3	1.18	1.00–1.40	0.95	0.80–1.14	1.38	1.15–1.66	1.42	1.19–1.71
≥ 60	13.6	1.82	1.55–2.15	1.65	1.39–1.95	2.19	1.84–2.61	2.24	1.88–2.67
*Race (Ethnics)*									
White	11.2	1		1		1		1	
Black	3.3	1.63	1.36–1.95	1.45	1.20–1.74	1.55	1.28–1.86	1.52	1.26–1.83
Yellow	0.6	1.30	0.86–1.92	1.10	0.72–1.65	1.18	0.77–1.78	1.18	0.77–1.78
Brown	81.2	1.43	1.29–1.58	1.36	1.23–1.50	1.43	1.30–1.59	1.43	1.29–1.59
Indigenous	0.8	2.22	1.62–3.01	2.52	1.83–3.45	2.69	1.94–3.69	2.49	1.80–3.43
Ignored	2.9	1.93	1.60–2.31	2.04	1.69–2.46	2.13	1.76–2.57	2.11	1.74–2.55
*HIV*									
No	85.2	1		1		1		1	
Yes	14.8	2.43	2.25–2.61	2.27	2.10–2.47	2.88	2.65–3.14	2.92	2.69–3.18
*Use of alcohol, tobacco or other drugs*									
No	83.2	1		1		1		1	
Yes	16.8	2.24	2.09–2.40	1.99	1.84–2.14	2.11	1.95–2.27	2.07	1.92–3.18
*Diabetes*									
No	90.2	1		1		1		1	
Yes	9.8	0.73	0.66–0.81	0.80	0.72–0.90	0.85	0.75–0.95	0.85	0.75–0.95
*Mental Illness*									
No	98.8	1		1		1		1	
Yes	1.2	1.53	1.19–1.95	1.33	1.03–1.71	1.30	1.00–1.68	1.27	0.98–1.64
*Other diseases*									
No	93.2	1		1		1		1	
Yes	6.8	1.46	1.31–1.62	1.60	1.43–1.79	1.63	1.45–1.82	1.61	1.44–1.81
*Clinical presentation*									
Extrapulmonary	14	1		1		1		1	
Pulmonary	81.8	0.96	0.89–1.05	0.93	0.85–1.03	1.10	1.00–1.21	1.07	0.97–1.18
Mixed (Pulmonary + Extrapulmonary)	4.2	1.74	1.50–2.01	1.14	0.97–1.33	1.30	1.11–1.52	1.29	1.10–1.51
*Bacilliferous*									
No	38.8	1		1		**–**	**–**	**–**	**–**
Yes	61.2	0.94	0.89–1.00	1.01	0.94–1.08	**–**	**–**	**–**	**–**
Programmatic vulnerability									
*HIV test performed*									
Yes	73.6	1				1		1	
No	26.4	1.51	1.42–1.61			2.22	2.07–2.38	2.24	2.08–2.40
*Directly Observed Treatment*									
Yes	12.7	1				1		1	
No	87.3	1.08	0.99–1.18			1.10	1.01–1.21	1.12	1.02–1.23
*X-ray*									
Yes	77.6	1				–		–	
No	22.4	1.01	0.94–1.08			–	–	–	–
*Diagnostic examination (bacilloscopy or molecular test)*									
Yes	86.6	1				1		1	
No	13.4	1.23	1.14–1.34			1.27	1.16–1.40	1.27	1.15–1.40
Social vulnerability									
*Special population*									
No	92.8	1						1	
Yes	7.2	1.63	1.47–1.81					1.52	1.36–1.69
*Education (years of schooling)*									
Illiterate	2.1	1						–	
1–4 years	14.6	0.61	0.50–0.74					–	–
5–9 years	19.9	0.64	0.53–0.78					–	–
More than 10 years	38.5	0.41	0.34–0.50					–	–
Ignored	19.8	0.80	0.66–0.97					–	–
Not applicable	5.1	0.30	0.29–0.46					–	–
*Beneficiary of government cash transfer program*									
No	51	1						1	
Yes	95	1.19	1.05–1.36					1.39	1.21–1.59
**Akaike criterium**				AIC1	26757	AIC2	26240	AIC3	26168

Source: Notifiable Diseases Information System (SINAN), Manaus Municipal Health Department.

Note: *OR - Odds ratio; CI - Confidence interval; Model 1 - multivariable logistic regression analysis considering a block of individual vulnerability variables; model 2 - multivariable logistic regression analysis considering a block of individual vulnerability variables and a block of programmatic vulnerability variables; model 3 - multivariable logistic regression analysis considering a block of individual vulnerability variables, a block of programmatic vulnerability variables and a block of social vulnerability variables. Other conditions represent any other condition reported or identified in the patient and recorded as other on SINAN, such as arthritis, arthrosis and systemic arterial hypertension. Special population includes the homeless population, the population deprived of their liberty, health professionals, immigrants and the population living in institutions.

When adjusting model 1 (proximal block with individual vulnerability variables), the following factors increase the chance of unsuccessful treatment: male sex, age between 15 and 49 and over 60 non-white race, HIV positive, use of alcohol, tobacco or other drugs, mental illness and mixed clinical form. In model 2, by adding a medial block with programmatic vulnerability variables, the same individual factors increase the chance of unsuccessful treatment, especially for all age groups and HIV positivity; in addition, the following programmatic variables increase the chance of non-success: not being tested for HIV, not undergoing DOT and not undergoing a diagnostic test. When formulating model 3, adding the distal block with social vulnerability variables, the same individual and programmatic factors increase the chance of unsuccessful treatment, adding the social vulnerability variables: being a special population and being a beneficiary of the government cash transfer program. It should be noted that in all three models, diabetes reduces the chance of unsuccessful treatment. The reduction in variance between the models was statistically significant (P-value < 0.001), indicating that the best fit is with model 3.

## DISCUSSION

The vulnerabilities experienced by patients are associated with unsuccessful TB treatment. Looking at the vulnerability factors associated with unsuccessful treatment in isolation shows their influence on the outcome in question, confirming what is pointed out in the literature, that TB is a disease linked to factors associated with vulnerabilities. These vulnerabilities are therefore also associated with the outcomes that TB patients can develop, leading to unsuccessful treatment^(15)^.

However, the multivariable analysis, in blocks of vulnerability, indicates that coping with unsuccessful TB treatment is a complex phenomenon, resulting from the multiplicity of simultaneous factors^(16)^. This shows that the interdependence between factors and vulnerabilities goes beyond the linear approach to TB management, suggesting the need to broaden the understanding of the influence of vulnerabilities in order to effectively resolve the situation of individuals and the disease in the municipality, taking into account the particularities^(14)^.

Individual vulnerability is the factor most strongly associated with unsuccessful TB treatment. This analysis identified that individual vulnerability factors should be looked at together, considering that unsuccessful TB treatment is multifactorial. Although the individual vulnerability factors identified here have been pointed out separately in other studies^(3,17)^. The interdependence of the factors suggests that assessing the patient holistically, with their multiplicities, can be more effective in controlling TB. It is up to professionals to change their outlook and focus on the individual with the potential to be cured, rather than thinking that the patient has the potential to non-success. Studies have shown that a positive and enlightened attitude allows for more assertive actions and also reinforces patient adherence to treatment^(18,19)^. Also of note in the individual process is the patients’ autonomy in managing their own treatment. For various reasons, such as cultural, religious or concerns about side effects, patients may point to these as factors for refusing to take medication. Recognizing and respecting this autonomy is fundamental to building trust and bonds in the ­patient-professional relationship. This type of therapeutic relationship promotes a humanized approach and can facilitate the patient’s commitment to treatment^(20)^. This is in line with the thinking that opposes blaming the patient for the loss of follow-up to TB treatment, but rather considering that this loss is also the responsibility of the service and a result of the context of vulnerability in which the subject is inserted^(4)^.

The greater strength of association (OR ≥ 2.0) of individual vulnerability indicates that being aged between 20 and 29, being of indigenous or unknown race, being co-infected with HIV and using alcohol, tobacco or other drugs should be points of attention, especially when considering that these factors are already adjusted for the other blocks of vulnerability.

In cases of TB/diabetes comorbidity, there was a reduction in unsuccessful TB treatment. It is known that diabetes is a disease associated with TB and worsens its prognosis^(21,22)^. However, the presence of this comorbidity with TB is associated with a reduction in cases of non-success, making it a protective factor; an identical result was obtained in an analysis of treatment default in Amazonas, between 2005 and 2010^(23)^. One relevant aspect that may explain this phenomenon is the awareness of self-care observed in patients with diabetes. Individuals diagnosed with diabetes are often advised on how to care for and manage their own health, which sometimes includes monitoring blood glucose levels, changing lifestyle habits and adhering to medication. This awareness of self-care among patients with diabetes can help with adherence to TB treatment. In addition, a study in Manaus shows that the program carried out with people with diabetes has both adherence and good results, although it has flaws. It is likely that the combined action of the programs on the patient with the comorbidity of TB and diabetes will minimize the loss of diagnosis and follow-up, reducing the chances of unsuccessful treatment^(24,25)^.

In the modeling process, programmatic vulnerability (model 2, Table 2) increased the strength of association of the individual vulnerability block, indicating an increase in the chance of unsuccessful TB treatment. However, there was no change in the estimates for model 3 when the social vulnerability block was added. This may indicate the extent to which the activities of the TB control program can influence the outcome studied. In this way, it increases the responsibility of the health service to provide more comprehensive care for TB patients, also taking into account their individual aspects.

Faults in the following procedures: taking the HIV test, or the DOT or the bacteriological diagnostic test (sputum smear microscopy or molecular test), are indicatives of compromised follow-up of the patient by the program. A study carried out in Manaus points to the importance of Primary Care in the success of TB treatment and identifies the distance between the service and diagnosing and monitoring cases properly as a cause of unsuccessful treatment^(2)^. The fault of the municipal service to diagnose and monitor cases properly determines programmatic vulnerability and this, in turn, influences the chances of unsuccessful treatment.

In the social vulnerability block, the findings indicate that being a special population and being a beneficiary of the government’s cash transfer program increase the chance of unsuccessful TB treatment. It is suggested that this is due to the social situation of vulnerability faced by these individuals, i.e. they have other related social fragilities. Studies corroborate that such populations have a mutual influence of the vulnerabilities assessed, determining the non-success^(26,27,28)^.

With regard to education, it is worth noting that in the bivariate analysis, an association with cure was observed for individuals with a higher level of education (+10 years), while unsuccessful outcomes were associated with lower levels of education or ignored data. A study corroborates the relationship between high education and favorable outcomes, as it reflects an improvement in economic and social status^(5)^. Data in the ignored category hinders a more accurate analysis, as well as showing a lack of care in terms of properly filling out the information system, which may also be a reflection of the care the patient receives.

This study has the limitation of using secondary data and restrictions on exploring more variables pertinent to the concept of vulnerability and its forms of presentation. In addition, the method of grouping vulnerabilities into blocks was a didactic and heuristic choice, which could be the subject of further analysis to arrive at the grouping that best represents the vulnerabilities. However, this methodological choice has allowed us to take a fresh look at the problem of unsuccessful TB treatment. Therefore, the need to observe vulnerabilities shows that the problem must be dealt with in a multifactorial way, taking vulnerabilities into account, in order to identify causes and propose solutions.

## CONCLUSION

It was observed that vulnerabilities and their components are multifactorial and associated with multifactorial outcomes in TB treatment. The main contribution of this analysis is to go beyond the factors associated with unsuccessful treatment in isolation, and to understand the interconnection of vulnerabilities, as well as the greater influence of individual vulnerability on the ­non-success, followed by programmatic vulnerability. This points to the need for a more equitable approach to monitoring TB patients. One strategy for dealing with patients’ vulnerabilities and needs is to adopt a line-of-care approach to TB care in health services, as recommended by the Brazilian Ministry of Health. Therefore, attention is drawn to the responsibility of the TB control program, whose programmatic vulnerability block is the one over which it has the most governance, and which in turn has an influence on individual vulnerability.
