# Supplementary material for: Factors associated with unsuccessful tuberculosis treatment in Manaus, Amazonas, from 2011 to 2021
Source: Rev Esc Enferm USP. 2024 Oct 7;58:e20240431. doi: 10.1590/1980-220X-REEUSP-2023-0431en (PMC11460654; doi:10.1590/1980-220X-REEUSP-2023-0431en)
Supplement: Translation

# Translation: Fatores associados ao insucesso do tratamento da tuberculose em Manaus, Amazonas, de 2011 a 2021

**DOI:** 10.1590/1980-220X-REEUSP-2023-0431pt

**Authors:** Felipe Alves de Almeida, Maria Jacirema Ferreira Gonçalves

**Affiliations:** 1 Universidade Federal do Amazonas, Escola de Enfermagem de Manaus, Programa de Pós-Graduação em Enfermagem, Manaus, AM, Brasil.; 1 Universidade Federal do Amazonas, Escola de Enfermagem de Manaus, Departamento de Enfermagem, Manaus, AM, Brasil.

**Keywords:** Tuberculose, Resultado do Tratamento, Adesão à Medicação, Vulnerabilidade em Saúde, Perda de Seguimento

## Abstract

**Objetivo:**

Identificar fatores de vulnerabilidade associados ao insucesso nos desfechos do tratamento da tuberculose no período de 2011 a 2021 em Manaus, Amazonas.

**Método:**

Estudo ecológico utilizando dados de notificação dos casos de tuberculose do Sistema de Informação de Agravos de Notificação, de 2011 a 2021, de residentes em Manaus. As variáveis referem-se aos desfechos do tratamento e à vulnerabilidade dos pacientes, conforme modelo teórico: individual, programática e social. Na análise testou-se associação entre vulnerabilidade e insucesso no tratamento de tuberculose, medido pela ocorrência de óbito, perda de seguimento do tratamento ou falência do tratamento. A estimativa de Odds Ratio com intervalo de confiança foi obtida por regressão logística, conforme modelo hierárquico.

**Resultados:**

Os seguintes fatores apresentam mais chance de insucesso no tratamento da TB: vulnerabilidade individual (faixa etária 20 a 29 anos e mais de 60 anos, raça indígena, HIV+, uso de drogas); vulnerabilidade programática (não realizar teste HIV), vulnerabilidade social (população especial).

**Conclusão:**

A vulnerabilidade individual foi mais fortemente associada ao insucesso. É necessária intervenção, explorando os pontos de maior vulnerabilidade individual, viabilizando uma atuação eficaz para evitar o insucesso no tratamento da tuberculose.

## INTRODUÇÃO

A Organização Mundial de Saúde (OMS) reconhece a gravidade da Tuberculose (TB) no mundo como problema de saúde pública e destaca 30 países que concentram cerca de 90% da carga da doença, dentre eles o Brasil. Em 2023, o Brasil registrou 80.012 casos novos de TB (37,3 casos por 100 mil habitantes) e 5.845 óbitos (2,8 óbitos por 100 mil habitantes) relativos ao ano de 2022. Nesse mesmo ano, o Amazonas notificou 3.548 casos novos (81,6 casos por 100 mil habitantes) e 218 óbitos (5,1 óbitos por 100 mil habitantes). Manaus registrou 2.512 casos novos (113,2 casos por 100 mil habitantes) e 134 óbitos (5,9 óbitos por 100 mil habitantes). Em 2023, o Amazonas apresentou a segunda maior incidência e a maior taxa de mortalidade por TB; Manaus destaca-se como a capital com maior incidência e a segunda maior taxa de mortalidade^(1)^.

Apesar do panorama crítico, a doença tem potencialidade de cura e seu tratamento tem sido alvo estratégico para seu controle. O acompanhamento do doente é realizado em nível de atenção primária e tem prognóstico para cura quando realizado corretamente^(2)^. Entretanto, é um tratamento longo, que pode causar reações adversas e, em alguns casos, pode haver dificuldades de acesso ao serviço de saúde, além de interferir em questões sociais e individuais do paciente, gerando possibilidades de insucesso no tratamento^(3)^.

O insucesso no tratamento aumenta a incidência dos casos, eleva riscos para resistência medicamentosa, aumenta chances de morbimortalidade e eleva custos para o setor público^(3)^. Neste estudo, o insucesso no tratamento inclui os seguintes desfechos: óbito, falência do tratamento e abandono do tratamento. Este último termo ainda é predominante na literatura e como tal registrado no sistema de informação, entretanto recomenda-se usar “perda de seguimento” do tratamento da TB, com o fim de incluir os diversos elementos envolvidos nesse desfecho, e de não culpabilizar o paciente como sujeito de abandonar o tratamento^(4)^.

Diversos fatores são apontados pela literatura como associados aos desfechos do tratamento da TB isoladamente. Os fatores associados à cura da TB são: sexo feminino, ter mais de 9 anos de estudo, raça branca, realizar Tratamento Diretamente Observado (TDO) e outros^(5)^. Quanto aos principais desfechos desfavoráveis, perda do seguimento e óbito por TB, são apontados como fatores associados idade maior ou igual a 60 anos, raça não branca, resultado HIV positivo, forma pulmonar, dentre outros^(6,7)^. Entretanto, em geral, esses fatores são observados na literatura de forma cartesiana, desconsiderando-se os cenários de vulnerabilidade que eles podem gerar, exercendo influência uns sobre os outros, e juntos, sobre o desfecho. Neste trabalho, entende-se o termo vulnerabilidade como a fragilidade de grupos ou indivíduos de ter suas necessidades atendidas e esta vulnerabilidade composta pela influência mútua de múltiplos fatores^(8)^.

Então, apesar do arcabouço teórico consistente construído ao longo dos anos sobre a TB, em Manaus o enfrentamento à doença ainda continua um desafio. Pontua-se que Manaus possui condições únicas em detrimento as demais localidades do país, em termos sociais e geográficos. Infere-se que essas condições podem interferir nos fatores que influenciam os desfechos do tratamento da TB, gerando cenários de vulnerabilidade próprios da localidade que necessitam ser estudados. Além disso, os estudos produzidos preocupam-se em avaliar os desfechos de maneira particionada, analisando o abandono do tratamento ou a cura de forma individualizada, e ignoram os demais desfechos^(9)^.

Sendo assim, com a proposta de entender os desfechos do tratamento da TB em Manaus, de forma a observar esse cenário com ótica mais integral e para procurar fornecer subsídios a saúde e enfermagem do Amazonas é que este trabalho objetiva identificar os fatores de vulnerabilidade associados ao insucesso nos desfechos do tratamento da tuberculose no período de 2011 a 2021, na cidade de Manaus, Amazonas.

## MÉTODO

### Tipo do Estudo

Estudo epidemiológico, observacional, ecológico analítico, com coleta retrospectiva das notificações anuais de casos novos de TB, do período de 2011 a 2021 em Manaus, Amazonas. Foram utilizadas as recomendações do checklist Strengthening the Reporting of Observational Studies in Epidemiology (STROBE), adaptadas ao tipo de estudo^(10)^.

### Local

Manaus, capital do Amazonas, possui dimensão de 11.401,092km^2^ e representa 52,5% da população do Amazonas, com uma estimativa de 2.255.903 habitantes, no ano de 2021. Em 2022, concentrou mais de 70% dos casos de TB do estado, sendo a capital com a segunda maior taxa de incidência de TB no Brasil^(11)^.

### População e Critérios de Seleção

Considerou-se como população de referência todos os casos novos de TB, de residentes em Manaus, notificados ao Sistema de Informação de Agravos de Notificação (Sinan), no período de 2011 a 2021. Como critério de inclusão adotou-se a seguinte seleção: caso novo, não sabe e pós-óbito, no tipo de entrada, todas as formas clínicas, com a condição de que os registros de encerramento estivessem completos no banco de dados, atualizado até dezembro de 2022. Foram excluídos da análise casos duplicados, mudanças de diagnóstico e transferências, devido à impossibilidade de avaliar corretamente o desfecho de insucesso no tratamento.

### Definição da Amostra

Considerou-se todo o universo de casos que preencheram os critérios acima, residentes em Manaus, notificados entre 2011 a 2021.

### Protocolo do Estudo

Foram utilizados dados secundários referentes às notificações de TB em Manaus extraídos do banco do Sinan municipal, os quais foram disponibilizados de forma anonimizada pela Secretaria Municipal de Saúde com atualização em maio de 2023, o que possibilitou análise de dados mais recentes.

Para a seleção das variáveis, foi utilizada ficha de notificação da TB e o conceito de vulnerabilidade^(8)^. O mesmo é expresso como a fragilidade de indivíduos ou grupos de ter suas necessidades atendidas, buscando elementos associados ou associáveis ao processo de adoecimento, não adoecimento e enfrentamento. Logo, segundo o conceito, o processo saúde-doença envolve a interação de um conjunto de aspectos, adotando-se a abordagem por dimensão de vulnerabilidade. Então, as variáveis independentes foram organizadas em blocos alinhados com as dimensões de vulnerabilidade:

– vulnerabilidade individual (características do indivíduo): sexo; faixa etária; raça; álcool, tabaco ou outras drogas; diabetes; doença mental; outros agravos (qualquer outra condição relatada ou identificada no paciente e registrada como outros no Sinan, tais como artrite, artrose e hipertensão arterial sistêmica); forma clínica e caso pulmonar bacilífero;
– vulnerabilidade programática (aspectos do programa de controle da TB): oferta do teste HIV; tratamento diretamente observado (TDO); oferta de raio-X de tórax; exame diagnóstico (baciloscopia de escarro ou teste rápido molecular);
– vulnerabilidade social (contexto social do indivíduo): vulnerabilidade social: população especial (população em situação de rua, população privada de liberdade, profissionais da saúde, imigrantes, população residente de instituições); escolaridade (em anos de estudos, que neste caso, considerou-se a faixa etária < 15 anos como ‘não se aplica’, pela impossibilidade de ter completado os estudos); e, beneficiário de programa de transferência de renda governamental.

A variável dependente considerou os desfechos do tratamento da TB: cura, perda do seguimento no tratamento da TB, óbito por TB, óbito por outras causas, demais desfechos (falência do tratamento, Tuberculose drogarresistente (TB-DR) e mudança de esquema) e insucesso do tratamento (perda do seguimento no tratamento da TB, óbito por TB, óbito por outras causas e falência do tratamento). Esses desfechos foram obtidos do campo “situação de encerramento” dos dados de notificação.

Foi incluída a variável “óbito por outras causas” no estudo devido a impossibilidade de averiguar qual seria o desfecho se os casos não tivessem morrido, no entanto eles teriam a mesma chance de qualquer um dos desfechos estudados. Outra questão que não se tem controle é se nos casos de preenchimento da declaração de óbito de fato, haveria a exclusão consciente de TB como causa do óbito, e sendo dado secundário, não há como verificar, haja vista que a vigilância do óbito por TB foi implantada em Manaus em 2017^(12)^. Ademais, a categoria ‘demais desfechos’ foi assim agrupada por dois fatores: baixa frequência e por serem geralmente decorrentes de interrupção prévia do tratamento. A variável insucesso foi agrupada da forma acima para se contrapor à cura, que seria o sucesso do tratamento, e ampliar a compreensão binária cura e perda de seguimento, visto que, na realidade há outros desfechos que necessitam ser analisados.

### Análise e Tratamento dos Dados

Todos os dados foram compilados e analisados através do software estatístico R versão 4.1.3 e no programa foram utilizados os pacotes: magrittr, readlx, tidyverse, dplyr, tidyr, lubridate, epiDisplay, foreign, r companion, carData e gtsummary. Na análise descritiva, foi apresentada frequência absoluta e percentual para as variáveis categóricas, explorando suas relações com o insucesso no tratamento da TB e com cada desfecho. Esse último aspecto foi analisado por meio do qui-quadrado de Pearson, considerando significância estatística < 5% e intervalo de confiança de 95%. A fim de identificar categorias de associação dentro de cada variável, foi realizada análise do resíduo padronizado do qui-quadrado, considerando significativos ao nível de 5% os resíduos >1,96. A magnitude de cada significância foi avaliada utilizando o Coeficiente V de Cramer, que permitiu quantificar a intensidade da relação entre o desfecho observado e o fator associado. Esse coeficiente varia entre 0 e 1, cujos valores mais próximos de 1 indicam mais intensidade do efeito.

Na análise logística bivariada foi testada relação entre as variáveis independentes e o desfecho de insucesso. Para análise de regressão logística múltipla foi realizada a modelagem hierárquica^(13)^, conforme a proximidade dos blocos de vulnerabilidade com o desfecho, considerando o modelo conceitual proposto. O modelo conceitual da vulnerabilidade embasa-se nos determinantes sociais da saúde, o qual determina proximidade de fatores de influência no desenvolvimento no processo saúde-doença. Os fatores proximais são inerentes aos indivíduos; os intermediários concernentes a vida e cotidiano; e, os fatores distais, aqueles referentes à macroestrutura econômica e governamental. Neste estudo os fatores individuais, programáticos e sociais, foram considerados respectivamente como proximais, intermediários e distais na modelagem hierárquica^(8,13,14)^. As variáveis que em análise bivariada obtiveram P-valor <0,20 foram agrupadas no respectivo bloco de vulnerabilidade; posteriormente, foram inseridas no modelo hierárquico considerando os blocos conforme proximidade teórica com o desfecho: vulnerabilidade individual, vulnerabilidade programática e vulnerabilidade social. O resultado está expresso pelo odds ratio (OR) com respectivo intervalo de confiança de 95%. A qualidade do modelo hierárquico foi avaliada pelo critério de informação de Akaike (AIC) e a seleção do modelo final foi realizada a partir da análise de redução da variância com P-valor < 0,05.

### Aspectos Éticos

O estudo foi submetido e aprovado pelo Comitê de ética em Pesquisa da Universidade Federal do Amazonas (CEP/UFAM) em 2023 sob o parecer 5.984.601, conforme Resolução n°466/12 do Conselho Nacional de Saúde (CNS), assegurando que os dados pertinentes a identidade dos sujeitos permanecerá em sigilo. Por ser pesquisa de dados secundários, o termo de consentimento livre e esclarecimento foi dispensado.

## RESULTADOS

Neste estudo, foi analisado 25.754 casos de TB notificados em Manaus de 2011 a 2021, que atenderam aos critérios de inclusão estabelecidos. A Tabela 1 apresenta a distribuição dos aspectos de vulnerabilidade e sua associação com os desfechos do tratamento da TB. Para as variáveis do bloco vulnerabilidade individual destacam-se as seguintes associações significativas para cada desfecho:

**Tabela 1:** Distribuição dos casos novos de Tuberculose de todas as formas, nos blocos de vulnerabilidade, conforme os desfechos do tratamento – Manaus, AM, Brasil, 2011 a 2021.

Variáveis	Cura 18640 (72,4)		Perda de seguimento do tratamento da TB 3987 (15,5)		Óbito TB 1415 (5,5)		Óbito por outras causas 846 (3,3)		Demais desfechos 866 (3,4)		Teste V-Cramer
	N	%	N	%	N	%	N	%	N	%	
Vulnerabilidade individual	(RP)		(RP)		(RP)		(RP)		(RP)		P-valor
*Sexo*											
Masculino	10.976	58,9	2.834	71,1	563	66,5	972	68,7	496	57,3	V = 0,099
	(–14,01)		**(13,51)**		**(3,06)**		**(5,71)**		**(2,6)**		**<0,001**
Feminino	7.664	41,1	1.153	28,9	283	33,5	443	31,3	370	42,7	
	**(14,01)**		(–13,51)		(–3,06)		(–5,71)		(–2,6)		
*Faixa etária (anos)*											
<15	1.039	5,6	154	3,9	28	3,3	41	2,9	15	1,7	V = 0,109
	**(7,37)**		(–3,47)		(–2,25)		(–3,67)		(–4,45)		**<0,001**
15–19	1.596	8,5	391	9,8	25	2,9	22	1,6	47	5,4	
	**(4,59)**		**(4,35)**		(–5,56)		(–9,27)		(–2,91)		
20–29	4.559	24,5	1.349	33,8	102	12,1	258	18,2	206	23,8	
	(–4,07)		**(13,77)**		(–8,92)		(–6,16)		(–0,93)		
30–39	3.647	19,6	931	23,3	104	12,3	337	23,8	191	22,1	
	(–4,3)		**(5,34)**		(–5,84)		**(3,45)**		(1,36)		
40–49	2.926	15,7	571	14,3	118	13,9	250	17,7	129	14,9	
	(1,36)		(–2,25)		(–1,27)		**(2,31)**		(–0,51)		
50–59	2.441	13,1	293	7,4	120	14,2	205	14,5	114	13,2	
	**(6,13)**		(–10,39)		(1,68)		**(2,55)**		(0,77)		
≥ 60	2.432	13	298	7,5	349	41,3	302	21,3	164	18,9	
	(–5,41)		(–12,54)		**(23,6)**		**(8,51)**		**(4,49)**		
*Raça*											
Branca	2.256	12,1	325	8,1	74	8,8	141	10	82	9,5	V = 0,034
	**(7,65)**		(–6,59)		(–2,28)		(–1,49)		(–1,62)		**<0,001**
Preta	588	3,1	160	4	22	2,6	47	3,3	24	2,8	
	(–1,62)		**(2,89)**		(–1,11)		(0,12)		(0,83)		
Amarela	106	0,6	24	0,6	1	0,1	8	0,6	1	0,1	
	(0,89)		(0,55)		(–1,71)		(0,11)		(–1,74)		
Parda	15.068	80,8	3.264	81,9	711	84	1.175	83	731	84,4	
	(–3,37)		(0,92)		**(2,05)**		(1,68)		**(2,36)**		
Indígena	128	0,7	43	1,1	10	1,2	15	1,1	9	1	
	(–3,2)		**(2,18)**		(1,28)		(1,15)		(0,82)		
Ignorada	484	2,7	171	4,3	28	3,3	29	2	19	2,2	
	(–3,53)		**(5,8)**		(0,77)		(–1,92)		(–1,22)		
*HIV*											
positivo	2.176	11,7	632	15,8	78	9,2	808	57,1	180	20,8	V = 0,290
	(–24,46)		(1,55)		(–4,82)		**(45,53)**		**(4,81)**		**<0,001**
negativo	16.464	88,3	3.355	84,2	768	90,8	607	42,9	686	79,2	
	**(24,26)**		(–1,55)		**(4,82)**		(–45,53)		(–4,81)		
*Uso de álcool, tabaco e outras drogas*											
Sim	2.553	13,7	1.122	15,8	190	22,5	326	23	226	26,1	V = 0,152
	(–23,81)		**(20,03)**		**(4,16)**		**(6,04)**		**(7,1)**		**<0,001**
Não	16.087	86,3	2.865	84,2	656	77,5	1089	77	640	73,9	
	**(23,81)**		(–20,03)		(–4,16)		(–6,04)		(–7,1)		
*Diabetes*											
Sim	1.948	10,4	254	6,4	118	13,9	119	8,4	125	14,4	V = 0,062
	**(4,29)**		(–8,22)		**(3,94)**		(–2)		**(4,48)**		**<0,001**
Não	16.692	89,6	3.733	93,6	728	86,1	1.296	91,6	741	85,6	
	(–4,29)		**(8,22)**		(–3,94)		**(2)**		(–4,48)		
*Doença Mental*											
Sim	192	1	42	1	27	3,2	28	2		0,7	V = 0,04
	(–2,28)		(–0,59)		**(5,69)**		**(3,03)**		(–1,27)		**<0,001**
Não	18.448	99	3945	99	819	96,8	1.387	98	860	99,3	
	**(2,82)**		(0,59)		(–5,69)		(–3,03)		(1,27)		
*Outros Agravos*											
Sim	1.152	6,2	226	5,7	150	17,7	171	12,1	136	15,7	V = 0,113
	(–9,54)		(–3,89)		**(12,19)**		**(7,46)**		**(9,98)**		**<0,001**
Não	17.488	93,8	3.761	94,3	696	82,3	1.244	87,9	730	84,3	
	**(9,54)**		**(3,89)**		(–12,19)		(–7,46)		(–9,98)		
*Forma Clínica*											
Pulmonar	15.369	82,4	3.415	85,7	647	76,5	918	64,9	672	77,6	V = 0,093
	**(5,56)**		**(7,15)**		(–3,93)		(–16,73)		(–3,11)		**<0,001**
Extrapulmonar	2.606	14	408	10,2	149	17,6	321	22,7	132	15,2	
	(–0,45)		(–7,53)		**(3,04)**		**(9,63)**		(1,04)		
Mista	665	3,6	164	4,1	50	5,9	176	12,4	62	7,2	
	(–9,81)		(–0,75)		**(2,28)**		**(15,39)**		**(4,15)**		
*Bacilífero*											
Sim	11.470	61,5	2.797	70,2	469	55,4	490	34,6	618	71,4	V = 0,153
	(0,07)		**(12,19)**		(–3,7)		(–21,39)		**(6,06)**		**<0,001**
Não	7170	38,5	1.190	29,8	377	44,6	925	65,4	248	28,6	
	(–0,07)		(–12,19)		**(3,7)**		**(21,39)**		(–6,06)		
Vulnerabilidade programática											
*Realização de teste HIV*											
Sim	14.110	75,7	2.667	66,9	438	51,8	1.100	77,7	654	75,5	V = 0,117
	**(12,05)**		(–10,54)		(–14,69)		**(3,59)**		(1,27)		**<0,001**
Não	4.530	24,3	1.320	33,1	408	48,2	315	22,3	212	24,5	
	(–12,05)		**(10,54)**		**(14,69)**		(–3,59)		(–1,27)		
*Tratamento Diretamente Observado (TDO)*											
Sim	2.417	13	438	11	165	19,5	154	10,9	34	3,9	V = 0,065
	**(4,02)**		(–3,06)		**(6,31)**		(–1,84)		(–7,73)		**<0,001**
Não	16.223	87	3.549	89	681	80,5	1.261	89,1	832	96,1	
	(–4,02)		**(3,06)**		(–6,31)		(1,84)		**(7,73)**		
*Raio-X*											
Sim	14.475	77,7	2.853	71,6	721	85,2	1.267	89,5	674	77,8	V = 0,094
	(0,23)		(–9,99)		**(5,4)**		**(11,07)**		(0,15)		**<0,001**
Não	4.165	22,3	1.134	28,4	125	14,8	148	10,5	192	22,2	
	(–0,23)		**(9,99)**		(–5,4)		(–11,07)		(–0,15)		
*Exame Diagnóstico*											
Sim	16.256	87,2	3.544	88,9	699	82,6	1.048	74,1	784	90,5	V = 0,096
	**(3,84)**		**(4,41)**		(–3,56)		(–14,41)		**(3,37)**		**<0,001**
Não	2.384	12,8	443	11,1	147	17,4	367	26,9	81	9,5	
	(–3,84)		(–4,41)		**(3,56)**		**(14,41)**		(–3,37)		
Vulnerabilidade Social											
*População especial*											
Sim	1.173	6,3	425	10,7	79	9,3	113	8	62	7,2	V = 0,063
	(–9,03)		**(9,22)**		**(2,46)**		(1,19)		(–0,04)		**<0,001**
Não	17.467	93,7	3.562	89,3	767	90,7	1.302	92	802	92,8	
	**(9,03)**		(–9,22)		(–2,46)		(–1,19)		(0,04)		
*Escolaridade*											
Analfabeto	320	1,7	75	1,9	64	7,6	50	3,5	29	3,3	V = 0,078
	(–6,76)		(–1)		**(11,32)**		**(3,91)**		**(2,64)**		**<0,001**
1 a 4 anos	2.663	14,3	583	14,6	162	19,1	220	15,6	133	15,4	
	(–2,33)		(0,04)		**(3,81)**		(1,03)		(0,64)		
5 a 9 anos	3.577	19,2	961	24,1	130	15,4	276	19,5	173	20	
	(–4,42)		**(7,29)**		(–3,34)		(–0,35)		(0,08)		
+ de 10 anos	7.680	41,2	1.208	30,3	188	22,2	501	35,4	393	45,4	
	**(13,28)**		(–11,86)		(–10,01)		(–2,63)		**(4,1)**		
Ignorado	3.359	18	1.004	25,2	274	32,4	327	23,1	123	14,2	
	(–11,3)		**(9,37)**		**(9,39)**		**(3,26)**		(–4,17)		
Não se aplica	1.041	5,6	156	3,9	28	3,3	41	2,9	15	1,7	
	**(7,3)**		(–3,35)		(–2,26)		(–3,7)		(–4,46)		
*Beneficiário do programa de transferência de renda governamental*											
Sim	889	4,8	205	5,1	66	7,8	81	5,7	43	5	V = 0,026
	(–2,58)		(0,49)		**(3,83)**		(1,31)		(–0,03)		**0,0013**
Não	1.775	95,2	3.782	94,9	780	92,2	1.334	94,3	823	95	
	**(2,58)**		(–0,49)		(–3,83)		(–1,31)		(0,03)		

Fonte: Sistema de Informação de Agravos de Notificação, Secretaria Municipal de Saúde em Manaus. Dados obtidos em 09/04/2023.

Notas: RP = Resíduo padronizado - A porcentagem soma 100% na coluna e os resíduos do qui-quadrado maior que 1,96 estão em negrito, correspondentes ao nível de significância para o excesso de ocorrências.

(1) cura: sexo feminino, faixa etária menor de 20 anos e 50 a 59 anos, raça branca, HIV negativo, não usar álcool, tabaco ou outras drogas, diabetes, ausência de doença mental ou outros agravos e, forma pulmonar;
(2) perda de seguimento do tratamento da TB: sexo masculino, faixa etária de 15 a 39 anos, raça preta, indígena ou ignorada, uso de álcool, tabaco ou outras drogas, ausência de diabetes ou outros agravos, forma pulmonar e bacilífero;
(3) óbito por TB: sexo masculino, faixa etária maior ou igual a 60 anos, raça parda, HIV negativo, uso de álcool, tabaco ou outras drogas, forma clínica pulmonar, extrapulmonar ou não bacilífera, e presença de diabetes, doença mental e outros agravos;
(4) óbito por outras causas: faixa etária a partir de 30 anos, HIV positivo, uso de álcool, tabaco ou outras drogas, doença mental e outros agravos, forma clínica extrapulmonar e mista, e casos não bacilíferos; e
(5) demais desfechos: faixa etária maior ou igual a 60 anos, raça parda, HIV positivo; uso de álcool, tabaco ou outras drogas, diabetes e outros agravos, forma clínica mista e casos bacilíferos.

No bloco vulnerabilidade programática observou-se as seguintes associações significativas:

(1) cura: realizar teste HIV, realizar TDO e realizar exame diagnóstico;
(2) perda de seguimento do tratamento da TB: não realizar teste HIV, não realizar TDO, não realizar raio-X e realizar exame diagnóstico;
(3) óbito por TB: não realizar teste HIV, realizar TDO, realizar raio-X e não realizar exame diagnóstico;
(4) óbito por outras causas: realizar teste HIV, realizar raio-X e não realizar exame diagnóstico; e
(5) demais desfechos: não realizar TDO e realizar exame diagnóstico.

Quanto ao bloco vulnerabilidade social as associações significativas, em cada desfecho, foram:

(1) cura: não ser população especial, escolaridade maior ou igual a 10 anos ou não se aplica, e não ser beneficiário do programa de transferência de renda governa mental;
(1) perda de seguimento do tratamento da TB: ser população especial, ter escolaridade entre 5 a 9 anos e escolaridade ignorada;
(3) óbito por TB: ser população especial, escolaridade analfabeta, inferior a 4 anos de estudo e escolaridade ignorada, e ser beneficiário do programa de transferência de renda governamental;
(4) óbito por outras causas: escolaridade analfabeta e escolaridade ignorada; e
(5) demais desfechos: escolaridade analfabeta e maior ou igual a 10 anos de estudo.

Os efeitos mais fortes estão nas variáveis: faixa etária, pessoas vivendo com HIV, usar álcool, tabaco ou outras drogas, outros agravos, casos bacilíferos e realização de teste HIV (Teste V de Cramer) (Tabela 1).

Outros agravos representam qualquer outra condição relatada ou identificada no paciente e registrada como outros no Sinan, tais como artrite, artrose e hipertensão arterial sistêmica. População especial inclui população em situação de rua, população privada de liberdade, profissionais da saúde, imigrantes, população residente de instituições.

Na Tabela 2 apresentam-se os resultados da análise de regressão logística bivariada e múltipla conforme o modelo hierárquico. Considerando as limitações, devido a possibilidade de interação e pelos problemas de preenchimento, a variável escolaridade não compõe o modelo múltiplo.

**Tabela 2:** Modelos de regressão logística simples e múltipla para fatores associados ao insucesso no tratamento da tuberculose, conforme blocos hierárquicos de vulnerabilidade individual, programática e social – Manaus, AM, Brasil, 2011 a 2021.

Variáveis	Análise bruta			Análise ajustada					
Vulnerabilidade individual				Modelo 1*		Modelo 2*		Modelo 3*	
	%	OR*	95%IC*	OR*	95%IC*	OR*	95%IC*	OR*	95%IC*
*Sexo*									
Feminino	38,4	1		1		1		1	
Masculino	61,6	1,62	1,52–1,72	1,36	1,28–1,45	1,37	1,28–1,46	1,37	1,28–1,46
*Faixa etária (anos)*									
<15	5,1	1		1		1		1	
15–19	8,2	1,28	1,07–1,53	1,19	0,99–1,43	1,76	1,46–2,13	1,81	1,50–2,20
20–29	25,2	1,75	1,50–2,04	1,33	1,14–1,57	2,03	1,72–2,40	2,04	1,72–2,41
30–39	20,2	1,76	1,50–2,06	1,22	1,03–1,44	1,82	1,53–2,16	1,84	1,55–2,19
40–49	15,5	1,5	1,27–1,76	1,10	0,93–1,30	1,60	1,35–1,92	1,63	1,37–1,95
50–59	12,3	1,18	1,00–1,40	0,95	0,80–1,14	1,38	1,15–1,66	1,42	1,19–1,71
≥ 60	13,6	1,82	1,55–2,15	1,65	1,39–1,95	2,19	1,84–2,61	2,24	1,88–2,67
*Raça*									
Branca	11,2	1		1		1		1	
Preta	3,3	1,63	1,36–1,95	1,45	1,20–1,74	1,55	1,28–1,86	1,52	1,26–1,83
Amarela	0,6	1,30	0,86–1,92	1,10	0,72–1,65	1,18	0,77–1,78	1,18	0,77–1,78
Parda	81,2	1,43	1,29–1,58	1,36	1,23–1,50	1,43	1,30–1,59	1,43	1,29–1,59
Indígena	0,8	2,22	1,62–3,01	2,52	1,83–3,45	2,69	1,94–3,69	2,49	1,80–3,43
Ignorada	2,9	1,93	1,60–2,31	2,04	1,69–2,46	2,13	1,76–2,57	2,11	1,74–2,55
*HIV*									
Não	85,2	1		1		1		1	
Sim	14,8	2,43	2,25–2,61	2,27	2,10–2,47	2,88	2,65–3,14	2,92	2,69–3,18
*Uso de álcool, tabaco e outras drogas*									
Não	83,2	1		1		1		1	
Sim	16,8	2,24	2,09–2,40	1,99	1,84–2,14	2,11	1,95–2,27	2,07	1,92–3,18
*Diabetes*									
Não	90,2	1		1		1		1	
Sim	9,8	0,73	0,66–0,81	0,80	0,72–0,90	0,85	0,75–0,95	0,85	0,75–0,95
*Doença Mental*									
Não	98,8	1		1		1		1	
Sim	1,2	1,53	1,19–1,95	1,33	1,03–1,71	1,30	1,00–1,68	1,27	0,98–1,64
*Outros Agravos*									
Não	93,2	1		1		1		1	
Sim	6,8	1,46	1,31–1,62	1,60	1,43–1,79	1,63	1,45–1,82	1,61	1,44–1,81
*Forma Clínica*									
Extrapulmonar	14	1		1		1		1	
Pulmonar	81,8	0,96	0,89–1,05	0,93	0,85–1,03	1,10	1,00–1,21	1,07	0,97–1,18
Mista	4,2	1,74	1,50–2,01	1,14	0,97–1,33	1,30	1,11–1,52	1,29	1,10–1,51
*Bacilifero*									
Não	38,8	1		1		–	–	–	–
Sim	61,2	0,94	0,89–1,00	1,01	0,94–1,08	–	–	–	–
Vulnerabilidade programática									
*Realização de teste HIV*									
Sim	73,6	1				1		1	
Não	26,4	1,51	1,42–1,61			2,22	2,07–2,38	2,24	2,08–2,40
*Tratamento Diretamente Observado*									
Sim	12,7	1				1		1	
Não	87,3	1,08	0,99–1,18			1,10	1,01–1,21	1,12	1,02–1,23
*Raio–X*									
Sim	77,6	1				–		–	
Não	22,4	1,01	0,94–1,08			–	–	–	–
*Exame Diagnóstico*									
Sim	86,6	1				1		1	
Não	13,4	1,23	1,14–1,34			1,27	1,16–1,40	1,27	1,15–1,40
Vulnerabilidade social									
*População especial*									
Não	92,8	1						1	
Sim	7,2	1,63	1,47–1,81					1,52	1,36–1,69
*Escolaridade*									
Analfabeto	2,1	1						–	
1 a 4 anos	14,6	0,61	0,50–0,74					–	–
5 a 9 anos	19,9	0,64	0,53–0,78					–	–
+ de 10 anos	38,5	0,41	0,34–0,50					–	–
Ignorado	19,8	0,80	0,66–0,97					–	–
Não se aplica	5,1	0,30	0,29–0,46					–	–
*Beneficiário do programa de transferência de renda governamental*									
Não	51	1						1	
Sim	95	1,19	1,05–1,36					1,39	1,21–1,59
Critério Akaike				AIC1	26757	AIC2	26240	AIC3	26168

Fonte: Sistema de Informação de Agravos de Notificação, Secretaria Municipal de Saúde em Manaus.

Nota: *OR – Razão de chance; IC – Intervalo de confiança; Modelo 1 – análise de regressão logística múltipla considerando bloco de variáveis de vulnerabilidade individual; modelo 2 – análise de regressão logística múltipla considerando bloco de variáveis de vulnerabilidade individual e bloco de variáveis de vulnerabilidade programática; modelo 3 – análise de regressão logística múltipla considerando bloco de variáveis de vulnerabilidade individual, bloco de variáveis de vulnerabilidade programática e bloco de variáveis de vulnerabilidade social. Outros agravos representam qualquer outra condição relatada ou identificada no paciente e registrada como outros no Sinan, tais como artrite, artrose e hipertensão arterial sistêmica. População especial inclui população em situação de rua, população privada de liberdade, profissionais da saúde, imigrantes, população residente de instituições.

No ajuste do modelo 1 (bloco proximal com variáveis de vulnerabilidade individual) os seguintes fatores aumentam a chance de insucesso do tratamento: sexo masculino, idade entre 15 e 49 anos e maior igual a 60 anos, raça não branca, HIV positivo, uso de álcool, tabaco ou outras drogas, doença mental e forma clínica mista. No modelo 2, ao acrescentar bloco medial com variáveis de vulnerabilidade programática, os mesmos fatores individuais aumentam a chance de insucesso do tratamento, com destaque para todas as faixas etárias e HIV positivo; além disso, as seguintes variáveis programáticas aumentam a chance de insucesso: não realizar teste para HIV, não realizar TDO e não realizar exame diagnóstico. Ao formular o modelo 3, acrescentando o bloco distal com variáveis de vulnerabilidade social, os mesmos fatores individuais e programáticas aumentam a chance de insucesso, adicionando-se as variáveis de vulnerabilidade social: ser população especial e ser beneficiário do programa de transferência de renda governamental. Destaca-se que nos 3 modelos, diabetes reduz a chance de insucesso do tratamento. A redução das variâncias entre os modelos foi estatisticamente significativa (P-valor < 0,001), indicando que o melhor ajuste se dá com o modelo 3.

## DISCUSSÃO

As vulnerabilidades vivenciadas pelos pacientes associam-se ao insucesso do tratamento da TB. Ao observar os fatores das vulnerabilidades associados ao insucesso do tratamento de forma isolada verifica-se a influência destes sobre o desfecho em questão, ratificando o que é apontado pela literatura, de que a TB é uma doença atrelada a fatores associados às vulnerabilidades. Tais vulnerabilidades, portanto, também estão associadas aos desfechos que o paciente com TB pode desenvolver, acarretando o insucesso do tratamento^(15)^.

Contudo, a análise múltipla, em blocos de vulnerabilidade, indica que o enfrentamento ao insucesso do tratamento da TB é um fenômeno complexo, decorrente da multiplicidade de fatores simultâneos^(16)^. Isso evidencia que a interdependência entre os fatores e as vulnerabilidades supera a ótica linear para o manejo da TB, sugerindo a necessidade da ampliação do entendimento da influência das vulnerabilidades para uma resolutividade eficaz do quadro do indivíduo e da doença no município, que considere as particularidades^(14)^.

A vulnerabilidade individual é o fator mais fortemente associada ao insucesso do tratamento da TB. Nesta análise identificou-se que os fatores de vulnerabilidade individual devem ser observados em bloco, considerando-se que o insucesso do tratamento da TB é multifatorial. Embora os fatores de vulnerabilidade individual aqui identificados sejam apontados em outros estudos separadamente^(3,17)^. A interdependência dos fatores, sugere que avaliar o paciente de forma integral, com suas multiplicidades pode ser mais efetivo no controle da TB. Aos profissionais, cabe mudar o olhar e se voltar para o sujeito com potencial de cura, ao invés de pensar que o paciente teria potencial de insucesso. Há estudos mostrando que a atitude positiva e esclarecida permite mais assertividade nas ações e também reforça a adesão do paciente ao tratamento^(18,19)^. Ademais, destaca-se ainda no processo do indivíduo, a autonomia do paciente sobre o manejo de seu próprio tratamento. Por diversas razões, tais como, culturais, religiosas ou preocupações com efeitos colaterais podem ser apontados pelos pacientes como fatores de recusa para da tomada das medicações. Reconhecer e respeitar essa autonomia é um ponto fundamental para geração de confiança e vínculo no relacionamento paciente-profissional. Esse tipo de relação terapêutica promove uma abordagem humanizada e pode facilitar o engajamento do paciente ao tratamento^(20)^. Isso vai ao encontro do pensamento que se opõe a culpar o paciente pela perda de seguimento do tratamento da TB, e sim, considerar que essa perda também é responsabilidade do serviço e decorrente do contexto de vulnerabilidade no qual o sujeito está inserido^(4)^.

A maior força de associação (OR ≥ 2,0) da vulnerabilidade individual indica que ter idade entre 20 a 29 anos, ser de raça indígena ou ignorada, ser coinfectado com HIV e fazer uso de álcool, tabaco e outras drogas devem ser pontos de atenção, especialmente quando se considera que tais fatores já estão ajustados para os demais blocos de vulnerabilidade.

Nos casos de comorbidade TB/diabetes verificou-se a redução do insucesso de tratamento da TB. Sabe-se que diabetes é uma doença associada à TB e que piora o seu prognóstico^(21,22)^. No entanto a presença desta comorbidade a TB está associada a redução dos casos de insucesso, sendo um fator protetor; resultado idêntico foi obtido na análise de abandono do tratamento no Amazonas, referente ao período de 2005 a 2010^(23)^. Um aspecto pertinente que pode explicar esse fenômeno é a consciência de autocuidado observada em pacientes com diabetes. Indivíduos diagnosticados com diabetes são frequentemente orientados quanto ao cuidado e gestão da própria saúde, o que por vezes inclui, monitoramento dos índices glicêmicos, mudança nos hábitos de vida e adesão da medicação. Tal conscientização sobre o autocuidado dos pacientes com diabetes pode se traduzir numa maior adesão ao tratamento da TB. Além disso, estudo em Manaus evidencia que o programa realizado com pessoas diabéticas tem boa adesão e resultados, embora tenha falhas. É provável que a união da ação dos programas sobre o paciente com a comorbidade TB e diabetes minimizem as perdas de diagnóstico e de acompanhamento, reduzindo as chances de insucesso no tratamento^(24,25)^.

No processo de modelagem, a vulnerabilidade programática (modelo 2, Tabela 2) aumentou a força de associação do bloco da vulnerabilidade individual, indicando aumentar a chance de insucesso do tratamento da TB. No entanto não se observa modificação nas estimativas sobre o modelo 3, quando se acrescenta o bloco da vulnerabilidade social. Isso pode ser um indicativo do quanto as atividades do programa de controle da TB podem influenciar no desfecho estudado. Desse modo, amplia a responsabilidade do serviço de saúde, em cuidar de forma mais abrangente do paciente de tuberculose, considerando também seus aspectos individuais.

A não realização dos procedimentos: teste para o HIV, TDO e exame diagnóstico bacteriológico (baciloscopia ou teste molecular) são indicativos de comprometimento no acompanhamento do paciente pelo programa. Estudo realizado em Manaus aponta a importância da Atenção Básica no sucesso do tratamento da TB e identifica o distanciamento do serviço em diagnosticar e acompanhar os casos adequadamente como causa de insucesso no tratamento^(2)^. A falha do serviço do município em diagnosticar e acompanhar da forma adequada, determina a vulnerabilidade programática e esta, por sua vez, influência as chances de insucesso.

No bloco de vulnerabilidade social, os achados apontam que ser população especial e ser beneficiário do programa de transferência de renda governamental aumentam a chance de insucesso no tratamento de TB. Sugere-se que o isso ocorre devido a situação social de vulnerabilidade enfrentada por esses indivíduos, ou seja, possuem outras fragilidades sociais relacionadas. Estudos corroboram que tais populações possuem influência mútua das vulnerabilidades avaliadas determinando o insucesso^(26–28)^.

Sobre a escolaridade, cabe destacar que na análise bivariada, observou-se associação com a cura para indivíduos com maior grau de instrução (+10 anos), enquanto os desfechos de insucesso foram associados às escolaridades mais baixas ou dados ignorados. Estudo corrobora a relação da alta escolaridade com desfechos favoráveis, pois reflete melhoria na situação econômica e social^(5)^. Os dados na categoria ignorada dificultam análise mais acurada, assim como pode evidenciar descuido na atenção à saúde com o adequado preenchimento do sistema de informação, o que pode também ser reflexo do cuidado que o paciente recebe.

Este estudo apresenta a limitação de ser realizado com dados secundários e possuir restrições na exploração de mais variáveis pertinentes ao conceito de vulnerabilidade e de suas formas de apresentação. Além disso, o método de agrupar as vulnerabilidades em blocos foi uma escolha didática e heurística, o que pode ser objeto de outras análises para se chegar ao agrupamento que mais represente as vulnerabilidades. Entretanto, esta opção metodológica permitiu novo olhar para o problema do insucesso do tratamento da TB. Portanto, a necessidade de observação das vulnerabilidades evidencia que se deve lidar com o problema de forma multifatorial, considerando as vulnerabilidades, para identificar causas e propor soluções.

## CONCLUSÃO

Observou-se que as vulnerabilidades e seus componentes se associam de forma multifatorial aos desfechos de insucesso do tratamento da TB. A principal contribuição desta análise é ir além dos fatores associados ao insucesso do tratamento de modo isolado, e compreender a interconexão de vulnerabilidades, bem como a influência maior da vulnerabilidade individual sobre o insucesso, seguida da vulnerabilidade programática. Isso aponta para a necessidade de uma abordagem mais equânime no acompanhamento dos pacientes com TB. Uma estratégia para lidar com as vulnerabilidades e necessidades dos pacientes é adotar a abordagem da linha de cuidado à pessoa com TB nos serviços de saúde, como recomenda o Ministério da Saúde. Portanto, chama-se atenção para a responsabilidade do programa de controle da TB, cujo bloco de vulnerabilidade programática é aquele sobre o qual mais tem governabilidade, e que por sua vez, tem influência na vulnerabilidade individual.
